# Scalable unitary computing using time-parallelized photonic lattices

**DOI:** 10.1515/nanoph-2025-0498

**Published:** 2025-12-10

**Authors:** Hyungchul Park, Beomjoon Chae, Hyunsoo Jang, Sunkyu Yu, Xianji Piao

**Affiliations:** Intelligent Wave Systems Laboratory, Department of Electrical and Computer Engineering, Seoul National University, Seoul 08826, Korea; Wave Engineering Laboratory, School of Electrical and Computer Engineering, 35010 University of Seoul, Seoul 02504, Korea

**Keywords:** photonic computing, resonator, buffer, scalability, photonic circuit, unitary operation

## Abstract

Exploiting alternative physical dimensions beyond the spatial domain has been intensively explored to improve the scalability in photonic computing. One approach leverages dynamical systems for time-domain computation, enabling universal and reconfigurable unitary operations. Although this method yields *O*(*N*) scaling in both device footprint and gate count, the required computation time increases by *O*(*N* ^2^), which hinders practical implementation due to limitations in quality factors and modulation speeds of optical elements. Here, we propose time-parallelized photonic lattices that achieve *O*(*N*) time scalability while preserving the *O*(*N*) spatial scaling. We devise a pseudospinor buffer operation that temporally stores the optical information, thereby enabling parallel unitary computation. The proposed method not only mitigates the requirement for high-quality factors but also provides robustness against a broad range of defects, demonstrating the feasibility of time-domain photonic computation.

## 1 Introduction

Parallelism is at the heart of modern computing technologies, as shown in graphic processing units to achieve improved scaling of computational speed [1], [2]. In implementing parallel computing, a fundamental requirement is to realize synchronization over multiple signal flows, which enables coordinated operations of processing units when executing specific tasks. Synchronization in electronic computing is achieved through buffer elements, such as latches and flip-flops.

To address critical limitations in programmable photonic circuits (PPCs) [3], [4], [5], [6], [7], [8], [9], [10], [11], [12] – *O*(*N* ^2^) scalability in device footprint and gate count [13], [14] – time-domain unitary computation using resonator lattices has been proposed, achieving *O*(*N*) spatial scalability [15], [16]. In this programmable photonic time circuit (PPTC), however, it is more challenging to enable synchronization than conventional PPCs due to different operation times between photonic gates. The resulting lack of synchronization enforces sequential rather than parallel gate operations, causing the operating time to increase by *O*(*N* ^2^). This scaling in the time domain requires optical elements with ultrahigh quality (*Q*) factors to store light throughout the computation. For example, it was expected that resonators with *Q* ∼ 10^8^ would be required even for *N* = 10 in conventional PPTC platforms [15]. Therefore, it is urgent to devise a suitable optical buffer [17], [18], [19] that is compatible with computing circuits, overcoming the difficulty of achieving optical bistability [20], [21], [22] necessary for memory functions.

Here, we propose a parallelized PPTC that employs the buffer element of pseudospinor states to synchronize different SU(2) gates. By exploiting the temporal and spatial programmability of PPTCs, the states of light at the target sites can be buffered by decoupling neighboring resonators through destructive interferences. We apply the buffering to compensate for variations in SU(2) gate latency, enabling synchronized SU(2) operations. The proposed synchronization scheme allows for the pristine implementation of the Clements design [14] with *O*(*N*) time complexity, which substantially lowers the required *Q*-factor and improves robustness against defects compared to the previous scheme [15]. This result enables a practical implementation of photonic computing for both classical and quantum applications.

## 2 Time-parallelized lattices

### 2.1 Synchronized PPTCs

Implementing high-dimensional and universal unitary operations using light signals [13], [14], [23], [24], [25], [26], [27], [28] has become an urgent issue in achieving photonic deep learning acceleration [29], [30] and quantum gate operations [7], [31], [32]. Although recent studies have enabled the use of higher-dimensional units for more compact design [23], [24], [25], [26], [27], [28], the most conventional approach for analytically deterministic design procedures relies on the factorization of a matrix *U*_*N*_ ∈ U(*N*) into a set of SU(2) matrices and a diagonal matrix [13], [14]. Each step of factorization is designed to achieve the nulling of an off-diagonal component, by applying a universal SU(2) operation to the unitary matrix *U*_*N*_. Such a nulling process continues sequentially until the matrix becomes diagonal, thereby allowing for the deterministic configuration of *U*_*N*_ through the cascaded multiplications of physically achievable SU(2) and diagonal matrices.

The sequence of the nulling processes can be implemented in different forms, for example, by nulling the components of each row (or column) sequentially – the Reck design [13] – or by nulling from higher- to lower-order off-diagonals – the Clements design [14]. These sequences lead to distinct circuit depth, as shown in the Clements design with a half of the circuit depth compared to the Reck design. However, both approaches eventually possess *O*(*N*) scaling in the circuit depth, leading to *O*(*N* ^2^) scaling in the overall device footprint.

**Figure 1:**
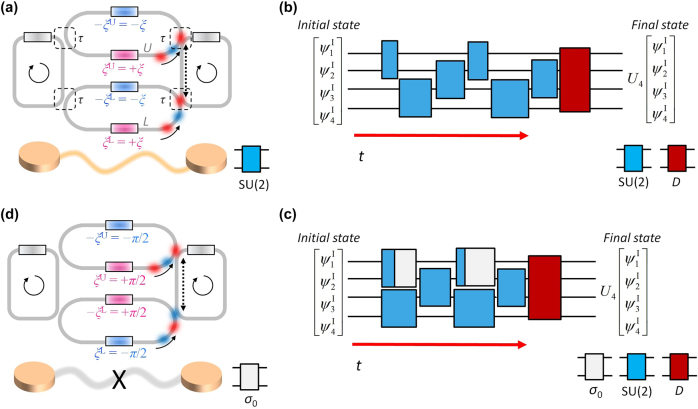
Synchronized operation of PPTC. (a) SU(2) gate composed of two resonators coupled via lower and upper loop couplers. Each loop coupler includes two phase shifters (colored boxes): ±*ξ* ^L^ and ±*ξ* ^U^ for lower and upper loop couplers, where +*ξ* ^L^ and +*ξ* ^U^ (or –*ξ* ^L^ and –*ξ* ^U^) denote the lower (or upper) phase shifters of each coupler, respectively. Each resonator includes the phase shifter for resonance perturbation Δ*ω*(*t*) (gray boxes). (b,c) Universal unitary operations *U*_*N*_ using the PPTC for *N* = 4: serial execution protocol (b) and parallel execution protocol using buffering (c). *D* ∈ U(4) denotes the designed 4-dimensional diagonal unitary matrix. (d) Buffering function achieved with the SU(2)-gate structure. In (a, d), dashed boxes and solid arrows denote the coupling regions and the direction of wave propagation, respectively. Dashed arrows represent the propagation length of an integer multiple of the effective wavelength of light.

To address the scalability issue, recent studies have proposed the utilization of wave evolutions along alternative physical axes, such as frequency synthetic dimension [33], [34], [35], [36] or temporal perturbations [15], [16], [37], [38], [39], [40]. In these approaches, one popular candidate is to utilize coupled-resonator optical waveguides (CROWs) [41], [42], [43], which enable dynamical modulation of the system for temporal- [15], [16] or frequency-domain [33], [35] tailoring of optical states. In the time-domain computing approach based on the concept of PPTCs [15], [16], the unit gate consists of a pair of resonators coupled through the zero-field loop couplers [42], [43] (Figure 1a), which can be modulated with refractive index changes. While each resonator supports the clockwise and counter-clockwise resonance modes, we focus on the clockwise mode, which leads to the Hamiltonian *H* governing the gate dynamics [15]:

(1) $$\begin{array}{ll} H & =-\omega_{0}\sigma_{0}-\frac{1}{2\tau }\left[\cos\xi^{\text{U}}\left(t\right)+\cos\xi^{\text{L}}\left(t\right)\right]\sigma_{x}\\ & \;-\frac{1}{2\tau }\left[\sin\xi^{\text{U}}\left(t\right)+\sin\xi^{\text{L}}\left(t\right)\right]\sigma_{y}-\Delta \omega \left(t\right)\sigma_{z}, \end{array}$$

where *ω*_0_ is the reference resonance frequency, Δ*ω*(*t*) is the resonance perturbation, *ξ* ^L^ (or *ξ* ^U^) characterizes the phase shifts of the lower (or upper) loop coupler, *τ* is the lifetime determined by the coupling between a resonator and a loop coupler, and *σ*_0_ and *σ*_*x*,*y*,*z*_ are the identity matrix and Pauli matrices, respectively (Figure 1a).

When we set Δ*ω*(*t*) = 0 and *ξ* ^L^ = *ξ* ^U^ = *ξ* (Figure 1a), the system Hamiltonian becomes *H* = −*ω*_0_ *σ*_0_ − (1/*τ*)[*σ*_*x*_cos*ξ* + *σ*_*y*_sin*ξ*] according to Eq. (1). Therefore, the gate yields an arbitrary and reconfigurable rotation operation on the Bloch sphere about the axis **n** = (cos*ξ*, sin*ξ*, 0), where the amount of the rotation is determined by the time Δ*t* maintaining the system parameters and the corresponding *H* [15]. For the modulation range of 0 ≤ *ξ* < 2*π*, the SU(2) gate operations required for decomposing arbitrary U(*N*) matrices are guaranteed by controlling Δ*t*.

These gate operations serve as the building blocks for realizing universal U(*N*) operations. To implement a given U(*N*) operation, we can construct a one-dimensional (1D) resonator lattice composed of the resonators coupled via the coupler in Figure 1a, while mapping each resonator mode to a basis. For an initial resonance state [*ψ*_1_^I^, *ψ*_2_^I^, *… ψ*_*N*_^I^]^T^, the cascaded, designed SU(2) gate operations for pairs of resonators (blue boxes in Figure 1b) implement the target matrix *U*_*N*_ ∈ U(*N*) with the final diagonal matrix (the red box in Figure 1b) that can be achieved with elementwise resonance detuning [15], [16]. We note that the SU(2) operations are executed sequentially in time [15], [16], in contrast to the spatial arrangement in the spatial PPCs [3], [4], [5], [6], [7], [8], [9], [10], [11], [12].

However, the critical hurdle of the PPTC described in [15], [16] is the poor scaling along the temporal axis, which exhibits *O*(*N* ^2^) scaling. This limitation originates from the operation principle of an SU(2) gate: the state rotation on the Bloch sphere of which the speed is determined by the coupling strength between resonators and loop couplers. Due to the static coupling determined by the stationary location of optical elements (dashed boxes in Figure 1a), the necessary times to achieve the target SU(2) operations are differentiated for each nulling process and the form of *U*_*N*_. Consequently, the temporal depths of SU(2) gates are not to be the same, leading to asynchronization and inevitably requiring sequential rather than parallel computing process (Figure 1b). The resulting *O*(*N* ^2^) temporal circuit depth not only degrades the operation speed but also enforces the necessity of ultrahigh *Q*-factor resonators to store light during the computation [15].

To resolve this poor time scaling issue, we develop the synchronization scheme for the PPTC. Figure 1c shows the proposed design protocol to resolve the time-scaling issue, by synchronizing multiple independent gate operations to the longest-latency one using the buffering functions (white boxes with 2 × 2 identity operations). This can be implemented by the odd-parity configuration with *ξ* ^U^(*t*) = −*ξ* ^L^(*t*) = *π*/2, which leads to decoupling between resonators through the system Hamiltonian *H* = −*ω*_0_ *σ*_0_ [15].

### 2.2 U(*N*) operation with buffering

**Figure 2:**
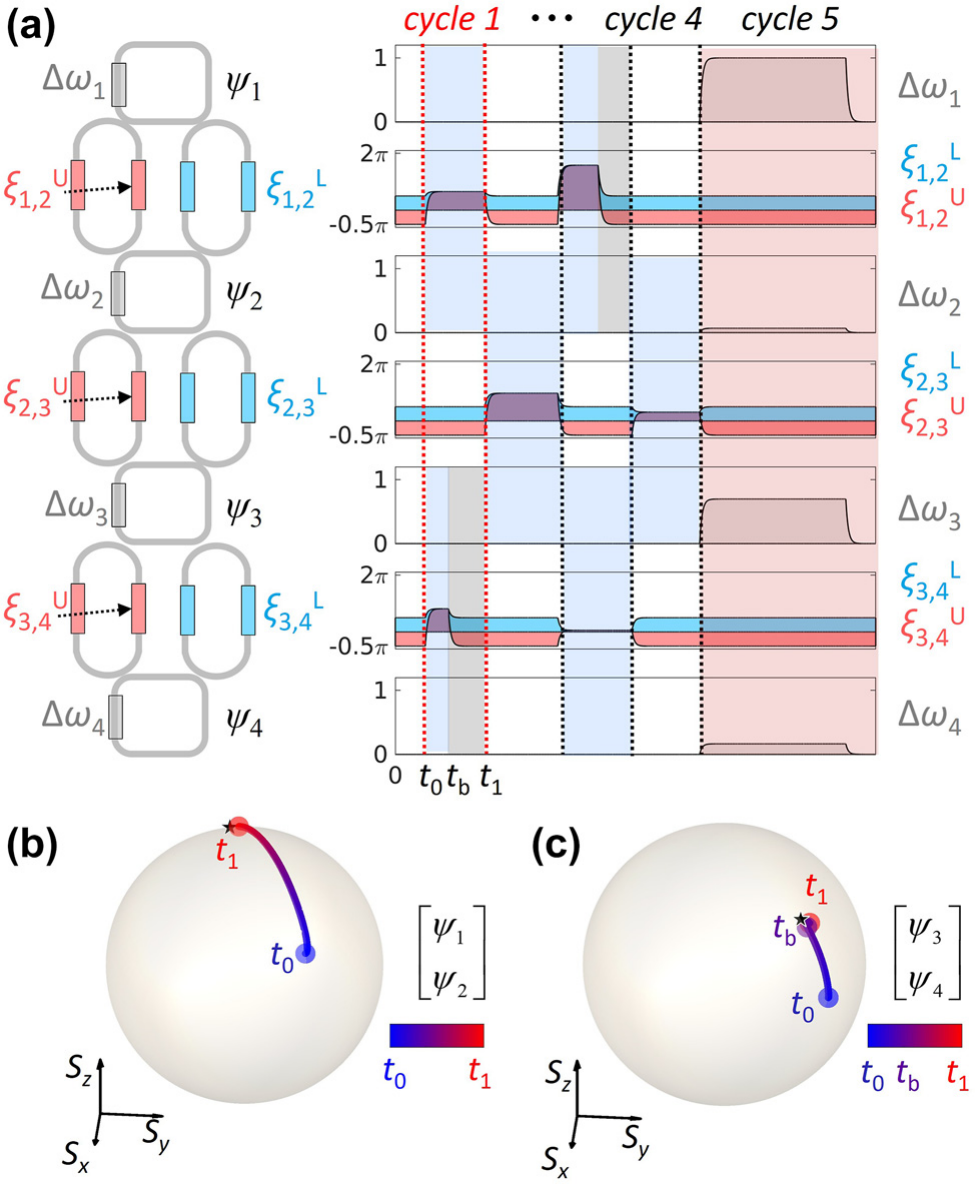
Synchronized *U*_4_ PPTC example. (a) Schematic of the PPTC and its time coded modulations: the resonance shift for the *n*-th resonator, Δ*ω*_*n*_, and the phase shifts *ξ*_*n*,(*n*+1)_^L,U^ for the loop coupler between the *n*-th and (*n*+1)-th resonators [15]. Each modulation is scheduled to satisfy synchronization. Considering the device bandwidth in real implementation, an ideal set of modulation signals are then low-pass filtered with the cut-off frequency *ω*_c_ = 0.01*ω*_0_. (b, c) Bloch-sphere representations of the pseudospinor states **ψ**_1,2_ = [*ψ*_1_, *ψ*_2_]^T^ (b) and **ψ**_3,4_ = [*ψ*_3_, *ψ*_4_]^T^ (c), which are defined by the resonance modes of the neighboring resonators 1 and 2, and 3 and 4, respectively. The Stokes parameters *S*_*i*∈*x*,*y*,*z*_ = **ψ**_*n*,(*n*+1)_^T^ *σ*_*i*_ **ψ**_*n*,(*n*+1)_ are plotted on the unit sphere. The evolutions of the states are plotted during time *t*_0_ ≤ *t* ≤ *t*_1_, where *t*_b_ denotes the completion time of the SU(2) operation between the resonators 3 and 4. Black stars denote the target states. The buffering function is observed in-between *t*_b_ (marked in purple) and *t*_1_ (marked in red).

To demonstrate the proposed scheme, we design the phase shifters of the PPTC under the protocol described in Figure 1c. As an example, we investigate the state evolution for a *U*_4_ matrix. The decomposition based on the Clements design for *U*_*N*_ [14] requires the circuit composed of (*N* + 1) layers: *N* layers for SU(2) operations and 1 diagonal-operation layer (Figure 2a). Each layer includes *n* (≈*N*/2) SU(2) gates in parallel, executing SU(2) matrices {*T*_1_, *T*_2_, … *T*_*n*_} during distinct operation times {Δ*t*_1_, Δ*t*_2_ … Δ*t*_*n*_}. Because Δ*t*_*i*_ ≠ Δ*t*_*j*_ in general, we introduce an additional buffer duration of max(Δ*t*_1_, Δ*t*_2_ … Δ*t*_*n*_) − Δ*t*_*i*_ for each operation *T*_*i*_ after completing the designed gate operation. This ensures that all gates in the set complete the operation synchronously with a common cycle period of max(Δ*t*_1_, Δ*t*_2_ … Δ*t*_*n*_).

Figure 2a illustrates the designed temporal modulation of the phase shifters under this protocol. In cycle 1, the gate operations *T*_1_ and *T*_2_ are executed in parallel, leading to the required SU(2) operations for the resonator pairs (1, 2) and (3, 4), respectively. Upon completion of *T*_2_ at time *t*_b_, the (3, 4) resonator pair is on the buffering mode, freezing the pseudospinor state without any energy exchange or the emergence of relative phase shift.

From the designed modulation, we calculate the PPTC operation following the same numerical method in [15], [16]: the application of the sixth-order Runge–Kutta (RK6) method [44] to solve the time-dependent equation *id*Ψ/*dt* = *H*Ψ with Eq. (1). The operations based on buffering are visualized on the Bloch sphere in Figure 2b and c. Because *T*_1_ has the longest latency execution time in this cycle, the designed SU(2) operation on [*ψ*_1_(*t*_0_), *ψ*_2_(*t*_0_)]^T^ completes at time *t*_1_ (Figure 2b). Meanwhile, the operation *T*_2_ completes earlier at time *t*_b_, and the state of [*ψ*_3_(*t*_b_), *ψ*_4_(*t*_b_)]^T^ remains stationary on the buffer mode until *t*_1_ (Figure 2c). As a result, the pseudospinor states of both pairs [*ψ*_1_(*t*_0_), *ψ*_2_(*t*_0_)]^T^ and [*ψ*_3_(*t*_0_), *ψ*_4_(*t*_0_)]^T^ reach their target output state simultaneously at *t*_1_, demonstrating the state synchronization via buffering.

## 3 Results

### 3.1 Time scaling

**Figure 3:**
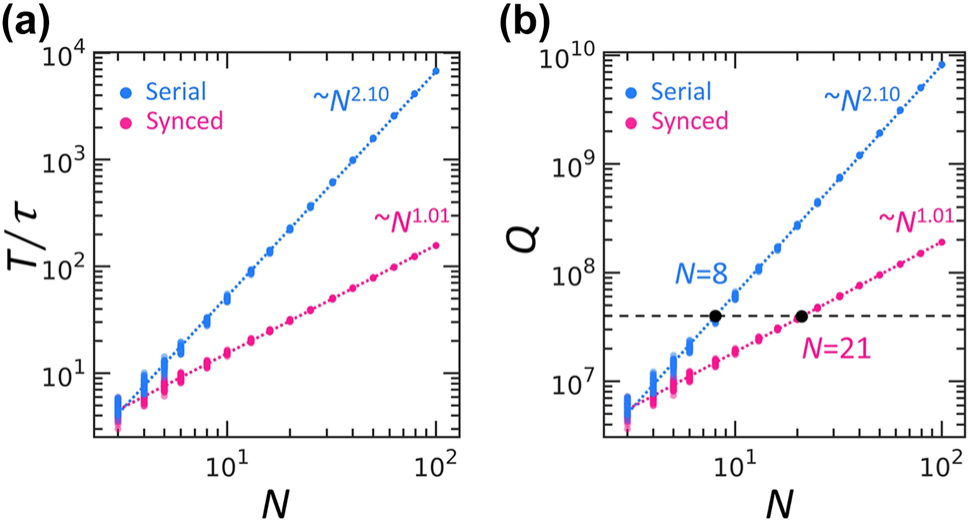
Time and *Q*-factor scaling. (a) Time scaling for *N*. The power-law exponents are *α* = 2.10 and *α* = 1.01 for the serial and synchronization schemes, respectively. (b) *Q*-factor scaling for *N*. In (a, b), blue and pink dots represent the 50 realizations for the serial and synchronization schemes, respectively. The colored dashed lines denote the power-law fittings of the data using the mean square error. The black dashed line in (b) corresponds to *Q* = 4 × 10^7^ experimentally demonstrated with the TFLN ring resonator [46]. The achievable *N* estimated with the fitted *Q* is marked as a black dot each for the serial and synchronization schemes.

To assess the impact of the proposed synchronization scheme, we evaluate the operation time *T* required to fully implement an arbitrary unitary matrix *U*_*N*_. Figure 3a presents the time scaling evaluated using 50 random Haar unitary matrices for each *N* [45], where the power-law fitting provides the exponent *α* = 2.10 for the serial scheme and *α* = 1.01 for the synchronization scheme. We observe an apparent improvement of time scaling from *O*(*N* ^2^) to *O*(*N*) under the proposed synchronization scheme, reflecting the reduction in required cycles from *M* = *N*(*N* – 1)/2 + 1 to *M* = *N* + 1 for *U*_*N*_ implementation.

Because our time-domain computation is conducted while light is stored in the resonators, reducing the operation time directly alleviates the requirement for high-*Q* optical elements. In Figure 3b, we estimate a lower bound of the intrinsic *Q* factor of each resonator via *Q* = *ω*_0_ *T*, where the full computation time *T* is obtained using the lifetime parameter *τ* = 10*T*_M_ to ensure the conservation of sufficient optical signals during the computation. We calculate the bound for 10 GHz modulation bandwidth of *T*_M_ ∼ 0.1 ns timescale, which is an accessible number with thin-film lithium niobate (TFLN) modulators [47]. The calculated *Q* in Figure 3b demonstrates the significant alleviation for implementing high-dimensional unitary operations under the synchronization scheme, enabling *U*_21_ operations with the *Q* = 4 × 10^7^ resonators, in contrast to the *U*_8_ limit in the prior serial scheme [15].

### 3.2 Fidelity analysis

Given the substantial reduction in operation time achieved with the synchronization scheme, we can reasonably expect improved operational fidelity at the same level of system defects, because shorter operation times reduce the exposure of light to defects. To examine this prediction, we evaluate various types of defects relevant to our architecture: fluctuations in the resonance frequency *ω*_0_ and the coupling lifetime *τ*, the altered zero-field condition of loop couplers, and resonator radiation loss. We characterize all these defects using a uniformly random distribution unif(*a*, *b*): *ω*_0_′ = [1 + unif(−*σ*, *σ*)]*ω*_0_, *ω*_0_″ = [1 + *i* × unif(0, 2*σ*)]*ω*_0_, 1/*τ*′ = [1 + unif(−*σ*, *σ*)]/*τ*, and Δ*φ* = unif(−*σ*, *σ*)*π*, where *ω*_0_′ and *ω*_0_″ denote the real- and complex-valued resonance frequencies accounting for resonance fluctuations and radiation loss, respectively, *τ*′ denotes the perturbed lifetime defined for the uniformly random perturbation of the coupling between a resonator and a loop coupler, and Δ*φ* is the unwanted phase shift exerted on each arm of the loop couplers.

For the target unitary operation *U*_*N*_ and its PPTC realization *V*_*N*_, we examine the operational fidelity with the following Frobenius-norm error:

(2) $$\varepsilon =\sqrt{\frac{\mathrm{Tr}\left[{\left(U_{N}-V_{N}\right)}^{†}\left(U_{N}-V_{N}\right)\right]}{N}},$$

where Tr[*A*] denotes the trace of matrix *A*. Although the error *ε* satisfies 0 ≤ *ε* ≤ 2 for the unitary output *V*_*N*_, this bound no longer applies to nonunitary *V*_*N*_. The defects characterized by the altered zero-field condition of loop couplers and resonator radiation loss lead to nonunitary *V*_*N*_ because optical energy is transferred into the loop coupler and environment, respectively, and therefore, the resonator-system Hamiltonian is no longer Hermitian.

To estimate *ε* statistically, we follow the procedure in the previous work [15]. In obtaining the *V*_*N*_ for one target *U*_*N*_, we calculate the PPTC operations for the *N* input states *ψ*_*n*_^I^ (*n* = 1, 2, …, *N*), which are obtained by normalizing *N* × 1 complex vectors that have the amplitudes unif(0, 1) and the phases unif(0, 2*π*). For initial field distributions of {*ψ*_*n*_^I^} inside the PPTC, we obtain *N* different outputs *ψ*_*n*_^O^ using the RK6 method, which allow for estimating *V*_*N*_ directly through the following inversion, as

(3) $$V_{N}=\left[\begin{array}{cccc} {\psi_{1}}^{\text{O}} & {\psi_{2}}^{\text{O}} & \ldots & {\psi_{N}}^{\text{O}} \end{array}\right]{\left[\begin{array}{cccc} {\psi_{1}}^{\text{I}} & {\psi_{2}}^{\text{I}} & \ldots & {\psi_{N}}^{\text{I}} \end{array}\right]}^{-1}.$$

In evaluating *ε*, we examine 10 random Haar unitary matrices as the target unitary operations.

**Figure 4:**
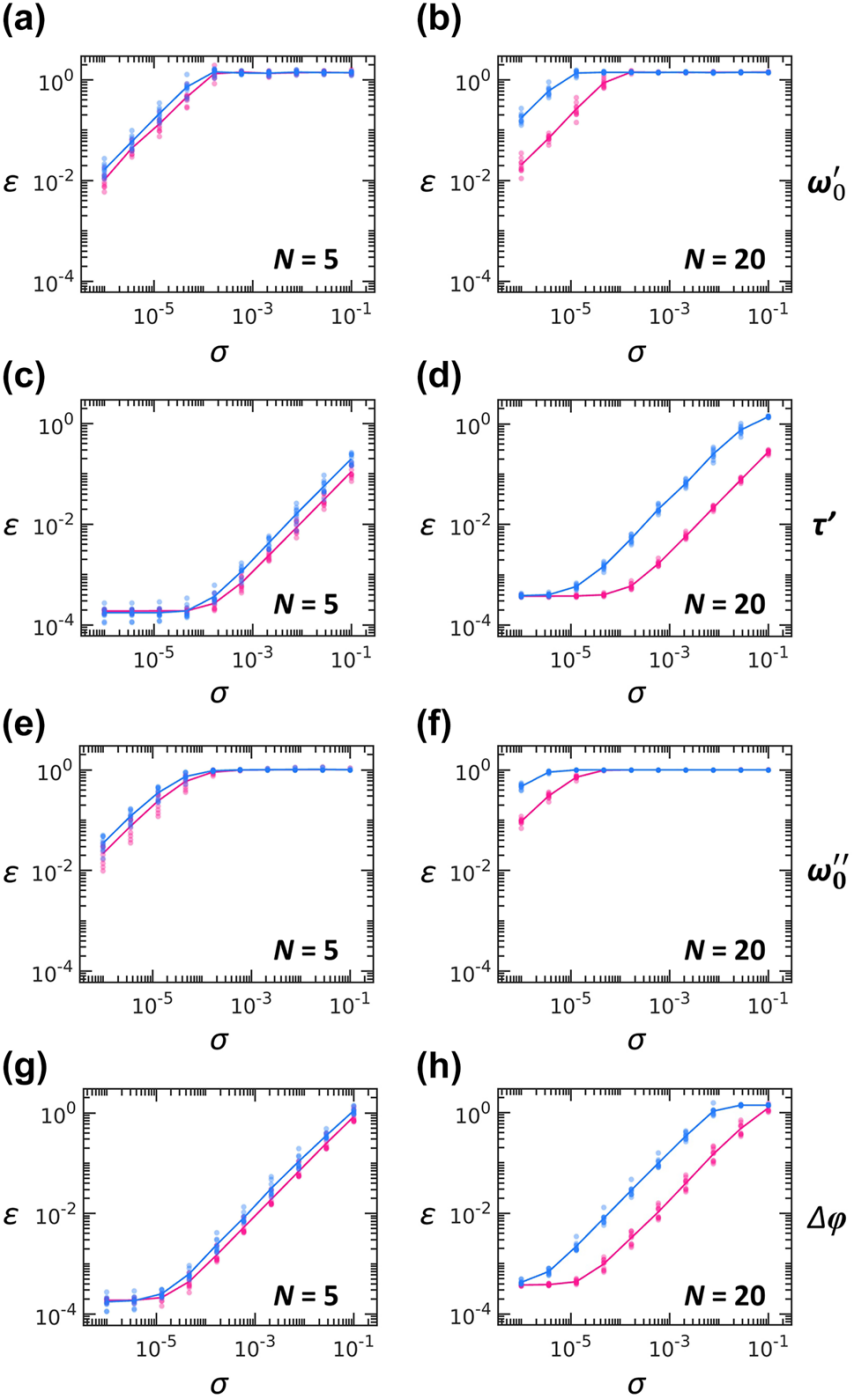
Robustness against defects. (a–d) Variations of errors with respect to the degree of defects *σ*: (a, b) resonance fluctuations, (c, d) lifetime fluctuations, (e, f) resonator radiation loss, and (g, h) phase-shift defects in loop couplers. Blue and pink dots denote *ε* obtained with 10 realizations of random Haar matrices at each value of *σ*. Solid lines denote their averages. We compare the serial (blue) and synchronization (pink) scheme for small- (*N* = 5) and large-scale (*N* = 20) systems for each type of defects.

Figure 4a–d shows the *σ*-dependent errors for the serial and synchronized implementations at *N* = 5 and *N* = 20. Although the detailed dependencies vary with the type of defects – for example, more sensitive responses to resonance perturbations than to the lifetime variations – the synchronization scheme consistently exhibits superior robustness against defects compared to the serial implementation. Notably, such an improvement in robustness by the synchronization is evident especially in larger systems (*N* = 20 in Figure 4(b, d, f, h) versus *N* = 5 in Figure 4(a, c, e, g)). This trend also holds for other values of *N* as shown in Supplementary Note S1.

### 3.3 Analysis for real implementation

We evaluate the computing performance of our PPTCs in terms of real implementation, including the comparisons with the prior serial configuration [15], [16] and with other electronic [48] and photonic [29] platforms. Our target calculation – *U*_*N*_ matrix multiplication to an *N* × 1 vector – includes *N* ^2^ multiplications and *N*(*N* − 1) additions of complex scalars. Because a multiplication and an addition of complex scalars require six and two operations regarding real-valued scalar calculations, respectively, the target calculation requires 8*N* ^2^ – 2*N* real-valued operations.

The operation times of the PPTCs are governed by the SU(2) gate operation time, which is comparable to *τ*. Because the time scalabilities of the serial [15], [16] and our synchronized implementations are *O*(*N* ^2^) and *O*(*N*), respectively (Figure 3), the operations (OPs) per second (OPS) of PPTC become ≈ (8*N* ^2^ – 2*N*)/(*N* ^2^ *τ*) ≈ 8/*τ* OPS in the serial design [15], [16] and ≈ (8*N* ^2^ – 2*N*)/(*Nτ*) ≈ 8*N*/*τ* OPS for our design. This estimation demonstrates that the parallelization enables scalable photonic computing with the operation speed proportional to the system size *N*. To reach the cutting-edge electronic GPU performance close to 10^3^ tera-OPS (TOPS) [48], the synchronized PPTC requires *N*/*τ* ≈ 10^14^, which can be achieved with 10^4^ resonators under ultrafast electro-optic modulation (*τ* ≈ 0.1 ns, [47], [49], [50]). Considering the difficulty of achieving the stable coupling of a huge number of resonators, the application of systolic matrix multiplications [51], [52], [53] can be a candidate using a lattice of about 10^2^ coupled resonators.

We also estimate energy efficiency, by assuming the modulator in [49], which supports 0.63 mW power consumption and 5 mm^2^ footprints. According to its *O*(*N*) scaling of the gate number, the PPTCs support ≈ 8,000/(*Nτ*) OP/J with the serial process [15], [16] and ≈ 8,000/*τ* OP/J with the synchronization design. Notably, our scheme using ultrafast electro-optic modulation surpasses the energy efficiency of the cutting-edge electronic GPU that supports a few TOP/J.

In terms of substituting the spatial axis with temporal axis, we further examine a comparison with the spatial PPCs [29]. We note that PPCs provide superior operation-speed scalability with ≈ (8*N* ^2^ – 2*N*)/*τ*_D_ OPS and comparable power efficiency ≈ 8,000/*τ*_D_ OP/J, where *τ*_D_ is the characteristic time of photodetection and the power efficiency is governed by the *O*(*N* ^2^) phase-shift bias power for large *N*. When assuming *τ*_D_ = 0.1 ns detection time, the PPC achieves 10 TOPS and 100 TOP/J with *N* ≈ 10, which leads to the footprint of 500 mm^2^ – comparable to the cutting-edge GPU die size of 10^3^ mm^2^. However, the PPC suffers from poor scalabilities in the footprint and gate number, leading to 5 × 10^4^ mm^2^ footprint and 10^4^ phase shifters for *N* ≈ 100, hindering the increase of computing dimension despite superior operation speed of 10^3^ TOPS. Therefore, the major advantage of the PPTC lies in the scalability of the gate number and footprint: 500 mm^2^ footprint and ∼500 phase shifters for *N* ≈ 100.

We note that the spatio-temporal computing circuit composed of waveguides and resonators, which has been very recently studied for the different purpose [54], [55] – nonlinear expressivity in photonic deep learning – would be a candidate platform resolving both the dimensionality limit of PPCs and the speed limit of PPTCs. This configuration also maintains the potential advantage of PPTCs as all-optical programmable devices – the use of resonance modes for energy localization and the consequent enhanced nonlinearity – which has been partly examined in [54], [55].

## 4 Conclusions

In conclusion, we proposed a synchronization scheme for the time-parallel assembly of gates in designing PPTC, enabled by a buffering function. Our analysis shows that the synchronization scheme reduces the temporal complexity from *O*(*N* ^2^) to *O*(*N*) while maintaining spatial efficiency. The resulting reduction in operation time significantly lowers the required resonator *Q*-factor, mitigating a critical hurdle for practical implementation of time-domain computation. We further explored the fidelity issue by comparing the serial and synchronized implementations, demonstrating that the proposed parallelization enhances robustness against various types of defects that are unavoidable in real fabrication processes.

Although we investigated the scalability of PPTCs based on the parallelization scheme, further issues, such as the input–output configuration [42], [43], physical origins of noises, and the synchronization of electro-optic modulation, need to be addressed for practical implementation. First, to excite and read out the signals with the PPTC platforms, one may consider the coupling of a probe waveguide to each resonator. In this case, there is a tradeoff relationship between the signal level and operation speed restricted by the ratio between the waveguide-resonator coupling *Q*-factor and the inter-resonator coupling *Q*-factor [15]. Therefore, our parallelization scheme, which substantially mitigates the restriction on the inter-resonator coupling *Q*-factor that must be much smaller than the intrinsic *Q*-factor of each resonator, extends the design space of system parameters for real implementation.

Second, while we examined the robustness of the system for the static form of defects, such as fabrication errors, possible major origins of the noise include the thermal crosstalk between optical components, which originates from dynamical modulation in phase shifters. In dense resonator meshes, the heat generated by one actuator perturbs the phase of many neighboring elements, leading to correlated phase errors [56], [57], [58], [59]. Although the parallel operation in our work may mitigate the influence of such a correlated noise in a statistical manner due to global change of refractive index over the entire system, this effect should be problem-specific and requires a further study based on multiphysics modeling [60].

Finally, when electro-optical modulation is incorporated into our PPTC design, time-varying voltage signals must be delivered to each phase shifter nearly simultaneously on a time scale much shorter than *τ*. Achieving this requires sub-nanosecond synchronization of the electrical signals, which can be realized using synchronized digital-to-analog converters together with delay precalibration [49]. However, when considering the accumulation of errors throughout the entire circuit, developing appropriate error-correction schemes [61], [62], [63], [64], [65] for our time-domain computation will become another critical research direction.

Our results establish a scalable and robust platform for high-dimensional photonic unitary operations, advancing toward the practical implementation of photonic computing and classical emulation of quantum phenomena. When considering recent efforts to increase the dimensionality of unit elements for more compact and scalable design of spatial PPCs [24], [25], [26], [27], [28], we can envisage further enhancement of scalability using two-dimensional lattice configuration of PPTCs with advanced parallelization schemes. The extension of the demonstrated scalable unitary operations to nonlinear functionalities [54], [55], [66], [67] would also be a future challenge.

## Supplementary Material

Supplementary Material Details
