# Supplementary material for: Scalable unitary computing using time-parallelized photonic lattices
Source: Nanophotonics. 2025 Dec 10;14(27):5527–35. doi: 10.1515/nanoph-2025-0498 (PMC12717889; doi:10.1515/nanoph-2025-0498)
Supplement: Supplementary file 1 — Supplementary Material Details [file j_nanoph-2025-0498_suppl_001.pdf]

Supplementary Information for "Scalable Unitary Computing using Time-Parallelized Photonic Lattices"

Hyungchul Park[1], Beomjoon Chae[1], Hyunsoo Jang[2], Sunkyu Yu[1†], and Xianji Piao[2*]

[1]Intelligent Wave Systems Laboratory, Department of Electrical and Computer Engineering, Seoul National University, Seoul 08826, Korea

[2]Wave Engineering Laboratory, School of Electrical and Computer Engineering, University of Seoul, Seoul 02504, Korea

E-mail address for correspondence: [†]sunkyu.yu@snu.ac.kr (S.Y.), *piao@uos.ac.kr (X.P.)

Note S1. Fidelity analysis on system size

Note S1. Fidelity analysis on system size

Figure S1 shows the scaling behaviours of errors ε with respect to N for different origins of system defects: resonance (Fig. S1a) and lifetime (Fig. S1b) perturbations, resonator radiation loss (Fig. S1c), and the altered zero-field condition of loop couplers (Fig. S1d). We observe scaling behaviours well approximated by power-law fitting (dashed lines in Fig. S1a-d). Notably, the synchronization scheme consistently yields superior fidelity as compared to the serial implementation.

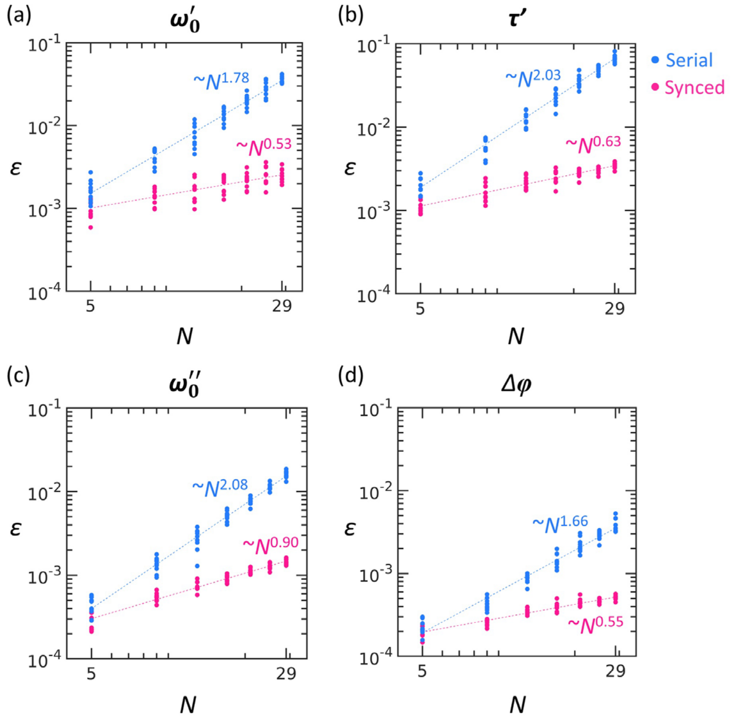

Fig. S1. Fidelity scaling. (a-d) Scaling of errors with respect to the system size N: (a) resonance fluctuations with $\sigma = 10^{-7}$, (b) lifetime fluctuations with $\sigma = 10^{-3}$, (c) resonator radiation loss with $\sigma = 10^{-8}$, and (d) phase-shift defects in loop couplers with $\sigma = 10^{-5}$. At each N, we examine 10 realizations of random Haar matrices. Dashed lines denote the power-law fitting. All the other parameters are the same as those in Fig. 4 in the main text.
